# P62 Links the Autophagy Pathway and the Ubiquitin–Proteasome System in Endothelial Cells during Atherosclerosis

**DOI:** 10.3390/ijms22157791

**Published:** 2021-07-21

**Authors:** SeJeong Kim, WoongJin Lee, KyoungJoo Cho

**Affiliations:** 1 College of Korean Medicine, Sangji University, Wonju 26339, Korea; nehemier@nate.com; 2 Department of Cognitive Science, Yonsei University, Seoul 03722, Korea; osang0616@yuhs.ac; 3 Department of Neurology, College of Medicine, Yonsei University, Seoul 03722, Korea; 4 Department of Life Science, Kyonggi University, Suwon 16227, Korea

**Keywords:** endothelial cell, atherosclerosis, autophagy, oxLDL, E3 ligase

## Abstract

Among autophagy-related molecules, p62/SQSTM1 is an adaptor for identifying and delivering intracellular cargo for degradation. Since ubiquitination is reversible, it has a switch role in autophagy. Ubiquitination is also involved in regulating autophagy in a timely manner. This study aimed to elucidate how p62-mediated autophagy is regulated in human endothelial cells and macrophages under atherosclerotic conditions, focusing on the lysosomal and proteasomal pathways. Co-cultured HUVECs and THP-1 cells were exposed to oxLDL (50 μg/mL) and autophagy was assessed. To downregulate p62, siRNA was administered, and the E3 ligases were inhibited by Heclin or MLN4924 treatment under the condition that cellular inflammatory processes were stimulated by oxLDL simultaneously initiated autophagy. Downregulating p62 induced an alternative degradation system, and the E3 ligases were found to be involved in the progression of atherosclerosis. Collectively, the present study demonstrated that the endothelial lipid accumulation under atherosclerotic conditions was caused by lysosomal dysfunction associated with autophagy.

## 1. Introduction

Endothelial dysfunction, as a pathological condition, is considered a crucial event in the progression of atherosclerosis. Endothelial cells (ECs) are arranged in many layers in large blood vessels, where they form a rigid wall, along with the connective tissue, and play an important role in regulating the function and structure of the vessel [1]. Endothelial dysfunction causes a lack of nitric oxide (NO), which can modulate leukocyte adhesion to the arterial walls [2]. Atherosclerosis is characterized by plaque in an arterial vessel where monocytes and ECs interact and respond to lipid overload [3]. Accumulated lipid in the arterial walls is one of the definitive features of atherosclerosis, and endothelial cell dysfunction in the inner wall of the vessel is an early event in atherosclerotic plaque formation [4,5].

Under physiological conditions, most cells activate compensatory mechanisms to handle harmful intracellular materials. Autophagy is a conserved cellular process that degrades and recycles damaged intracellular aggregates and organelles [6,7]. Successful autophagy generally contributes to cellular survival by acting against apoptosis and promoting cellular recovery by supplying biomaterials. In atherosclerotic conditions, autophagy is one of the responses to toxic intermediates found in atherosclerotic plaques, and autophagic processes concomitantly increase in macrophages [8]. Dysfunctional autophagy induces the accumulation of damaged mitochondria and the over-expression of the reactive oxygen species (ROS). In humans, autophagy also serves as a safeguard, for cells with atherosclerotic plaques, against cellular oxidative stress by polarizing mitochondria and preventing cytochrome *c* release [9]. Atherosclerosis progression is characterized by impaired autophagy. Autophagy deficiency in macrophages increases their susceptibility to foam cell formation, which plays an essential role in the atherosclerotic process. Various mechanisms have been reported to be involved in impaired autophagy, or autophagy-related molecular changes, during atherosclerosis [10]. ECs maintain the structure of thrombotic plaques, and autophagic cell death of ECs is detrimental to the structure of atherosclerotic plaques. The formation of atherosclerotic plaques is an acute clinical event that promotes the thrombosis of the atherosclerotic lesions. Given the stabilizing nature of plaques on rupture-prone lesions, the induction of autophagy in macrophages might be a promising strategy in plaques that are not obstructing the lumen, but are prone to rupture [11].

A well-known receptor for autophagy, p62/SQSTM1 (p62), plays an important role in shuttling protein aggregates from the cytosol to autophagosomes in selective autophagy, aggrephagy, and lipophagy [12]. Since p62 transports intracellular autophagic cargo, including lipids, to autophagosomes for degradation, the disruption of autophagy leads to p62 accumulation [13]. Increased levels of p62 are indicative of defective autophagic flux in various diseases [14,15,16,17]. As an adaptor, p62 consists of a nuclear localization signal (NLS), an export motif (NES), an N-terminal Phox-BEM1 (PB1) domain, an LC3-interacting region (LIR), a Keap1-interacting region (KIR), and a C-terminal ubiquitin-associated (UBA) domain [18,19]. The UBA domain of p62 interacts with ubiquitin or polyubiquitin chains at K48 and K63, and the LIR domain leads p62 to carry polyubiquitinated cargo to autophagy [20,21,22]. There are two major intracellular degrading systems: the ubiquitin-proteasome system (UPS) and the autophagy–lysosomal system. These systems serve a major surveillance role in detecting and removing misfolded proteins [23]. As p62 serves as a bridge, proteasome inhibition can activate autophagy [24]. Since ubiquitination is reversible, it has a bidirectional role in autophagy. Ubiquitination is also involved in regulating autophagy in a timely manner [25].

Although increased p62 levels under damaged cellular conditions have been reported to denote autophagy deficiency, it is thought that the accumulation of p62 can be a maladaptive or cytoprotective response, particularly in atherosclerosis. This study aimed to elucidate the role of p62-mediated autophagy in human ECs, under atherosclerotic conditions. Here, we showed that endothelial lipid accumulation during atherosclerosis was caused by lysosomal dysfunction, rather than proteasomal deficits.

## 2. Results

### 2.1. Innate Immune Responses and Inflammation Are Induced by oxLDL in Co-Cultured HUVEC and THP-1 Cells

**Figure 1:**
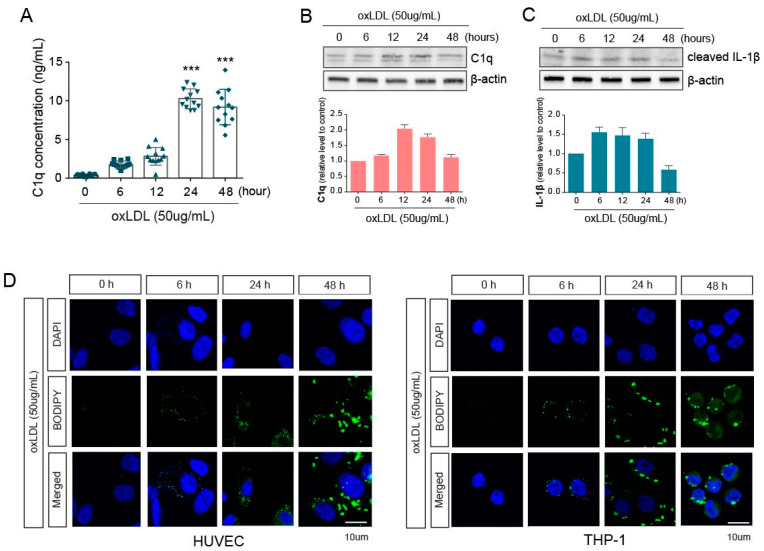
Exposure of oxLDL on co-cultured macrophages and endothelial cells. (**A**) C1q concentration in the supernatant collected from co-culture media was measured using ELISA. (**B**) The protein levels of C1q in HUVECs were assessed by western blotting in whole-cell lysates at several time points after oxLDL exposure. (**C**) The protein levels of cleaved IL-1β were analyzed by western blotting in THP-1 whole-cell lysates at several time points after oxLDL exposure. (**D**) The accumulated lipids in HUVEC (left) and THP-1 (right) cells were stained with BODIPY and were detected by fluorescence microscopy. ***, *p* < 0.0001.

To mimic vessel conditions under atherosclerotic conditions, we co-cultured human umbilical vein endothelial cells (HUVEC) and THP-1 cells and treated them with oxLDL (50 µg/mL) for 6, 12, 24, or 48 h. We then collected the supernatant from the co-culture and measured the levels of C1q, an innate immune molecule that is part of the classical pathway of the complement system, using ELISA. C1q increased until 24 h after oxLDL treatment and did not further increase (Figure 1A). Next, cell lysates, isolated from the co-culture, were analyzed at the protein level. Notably, C1q protein levels in HUVECs were similar to the secreted C1q levels (Figure 1B). Furthermore, we analyzed the cleaved IL-1β protein levels in THP-1 cells in response to oxLDL and found that IL-1β cleavage was the highest at 24 h after oxLDL treatment and slightly decreased at 48 h (Figure 1C). To investigate the lipid deposition following oxLDL treatment in each cell type, HUVECs and THP-1 cells were stained with BODIPY. The lipid loading by oxLDL treatment was detected at 0, 6, and 24 h in co-cultured HUVECs and THP-1 cells, with the lipid accumulation significantly increased at 24 h, rather than at 6 h (Figure 1D).

### 2.2. Autophagy Is Induced but Malfunctions in Atherosclerotic Conditions

**Figure 2:**
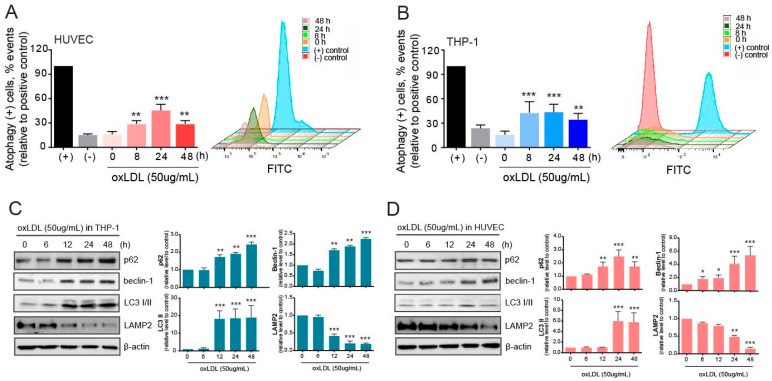
Initiation of autophagy in co-cultured macrophages and endothelial cells upon oxLDL exposure. (**A**) After oxLDL treatment, autophagy in HUVECs was evaluated using the CYTO-ID kit and presented as relative values to the positive control. (**B**) Autophagy in THP-1 cells after oxLDL administration was evaluated using the CYTO-ID kit and presented as relative values to the positive control. (**C**) Western blot analysis in THP-1 cells was used in the evaluation of autophagy-related molecules. (**D**) Autophagy-related molecules were evaluated by western blotting in HUVECs. *, *p* < 0.05; **, *p* < 0.001, ***, *p* < 0.0001.

Primarily, autophagy levels were determined using the CYTO-ID kit in HUVECs and THP-1 cells in the co-culture. The number of autophagy-positive HUVECs was significantly increased by 8 h, peaked at 24 h, and then decreased at 48 h after oxLDL exposure (Figure 2A). In THP-1 cells, the number of autophagy-positive cells increased at 8 h but remained at elevated levels after 48 h (Figure 2B). Autophagy-related molecules were also examined to measure the effect of oxLDL on autophagy in the THP-1 cells co-cultured with HUVECs after oxLDL exposure (Figure 2C). The expression levels of the autophagic adaptor p62 increased in a time-dependent manner upon oxLDL exposure. The levels of the autophagy-initiating molecule beclin-1 also increased, and the lapidated LC3 (LC3-II) was significantly augmented. These altered levels of autophagy molecules suggested that autophagy was initiated, selected intracellular cargo to be cleared, and executed in THP-1 cells. However, this study mainly focused on damaged HUVECs under atherosclerotic conditions derived from exposure to oxLDL. As shown in Figure 1D, we determined that lipids were loaded into the HUVECs and, thus, investigated the role of autophagy in clearing the deposited lipids in the HUVECs (Figure 2D). The aforementioned autophagy molecules in HUVECs were analyzed by western blotting, and the results showed that both beclin-1 and p62 protein levels were increased time-dependently, but slightly delayed, compared to the pattern in THP-1 cells. Moreover, the levels of LC3-II were increased after 24 h of oxLDL exposure but decreased by 48 h. Further, the LAMP2 protein levels were reversed to p62 protein level. Unexpectedly, the levels of LAMP2 were reduced in a time-dependent manner following exposure to oxLDL.

### 2.3. Downregulated p62 Induces Alternative Degrading Systems

**Figure 3:**
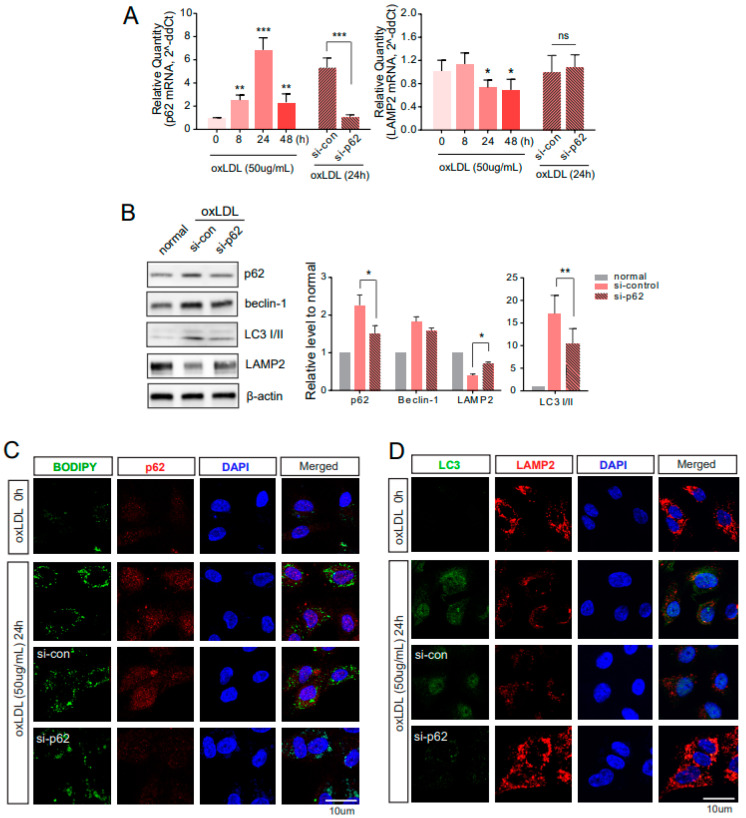
Autophagic dysfunction in HUVECs by downregulated p62. (**A**) Under atherosclerotic conditions derived by oxLDL exposure, the mRNA levels of p62 and LAMP2 were measured by real-time quantitative PCR. (**B**) After treatment with si-p62 for 24 h, cells were subsequently exposed to oxLDL for 24 h. The protein levels of each molecule were analyzed by western blotting (left), then quantified (right). (**C**) The consequences of downregulating p62 were evaluated with BODIPY staining for intracellular lipid and immunocytochemistry for p62. (**D**) Double-immunocytochemistry of LC3 and LAMP2 was done in si-p62 treated HUVECs under oxLDL exposure. ns, no significance; *, *p* < 0.05; **, *p* < 0.001, ***, *p* < 0.0001.

To better understand the role of autophagy and impaired autophagy under atherosclerotic conditions, p62 was silenced by siRNA treatment for 48 h prior to oxLDL exposure. Accordingly, when co-cultured with oxLDL for 24 h, the p62 mRNA (Figure 3A, left) and protein levels were downregulated in HUVECs treated with siRNA (Figure 3B). Prolonged exposure to oxLDL impairs autophagy in ECs. During autophagy, p62 plays a role as an adaptor for oxLDL. When p62 was silenced in the co-culture of the THP-1 and HUVECs, LC3-II in the HUVECs was reduced, and the loaded lipids correspondingly increased (Figure 3B). The formation of the p62 bodies was identified with clear punctate structures, and the loaded lipids stained by BODIPY were not reduced in each siRNA-treated group of HUVECs (Figure 3C). Immunocytochemistry also provided results similar to the western blot assays, showing LC3 signals with clear punctate structures that were significantly reduced in siRNA-p62 treated HUVECs (Figure 3D). Consistently, the LC3-positive levels representing autophagy execution were hampered by p62 downregulation and proteasomal system inhibitors. In contrast to the LC3 findings, the LAMP2 mRNA level was decreased by oxLDL exposure, and p62 downregulation did not significantly alter the LAMP2 mRNA level (Figure 3A, right). However, the protein level of LAMP2 increased by p62 downregulation (Figure 3B). Additionally, the LAMP2-positive signals in siRNA–p62-treated HUVECs were slightly augmented, which was sufficient to overcome oxLDL damage (Figure 3D).

### 2.4. E3 Ligases Are Involved in Autophagy under Atherosclerotic Conditions

**Figure 4:**
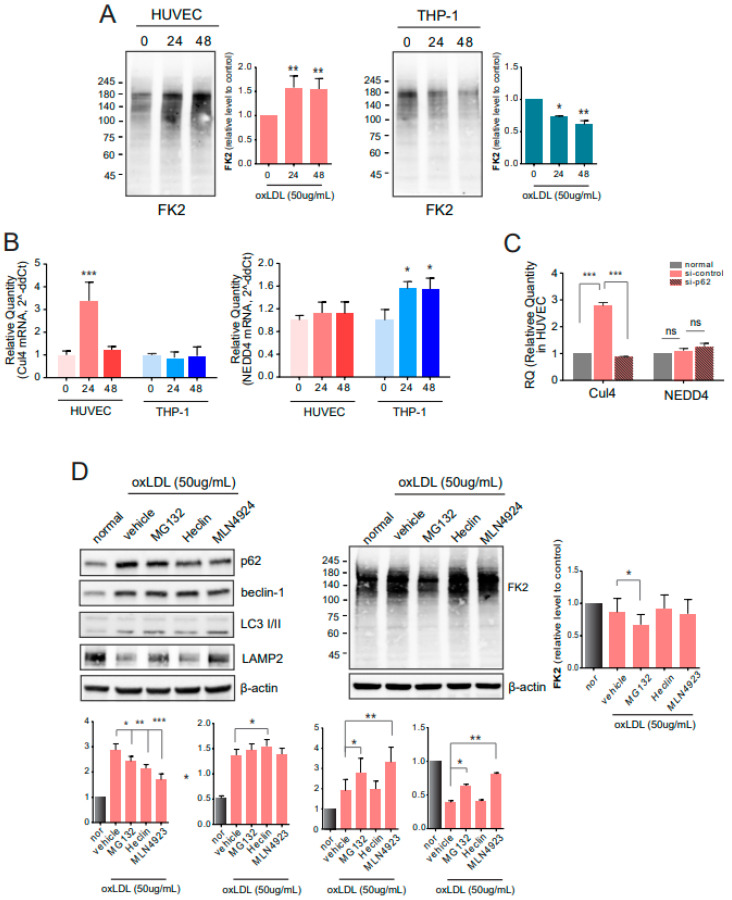
Involvement of E3 ligases in autophagy. (**A**) At several time points after oxLDL treatment, polyubiquitinated proteins in HUVECs and THP-1 cells were detected by western blotting with antibodies against FK2. (**B**) The qPCR for Cul4 and NEDD4 was performed in HUVECs and THP-1 cells. (**C**) After administering p62-siRNA to HUVECs, the mRNA levels were measured by qPCR. (**D**) Changes in autophagy-related molecules and polyubiquitinated proteins in HUVECs were assessed by western blotting (left) and quantified (right). ns, no significance; *, *p* < 0.05; **, *p* < 0.001, ***, *p* < 0.0001.

As shown in Figure 3, the altered pattern of autophagy-related molecules following the p62 downregulation represented lysosomal degradation of cargo, although the autophagy was initiated by oxLDL exposure. To investigate proteasomal degradation, we performed immunoblotting with antibodies against polyubiquitinated proteins (FK2) but not free ubiquitin. The levels of polyubiquitinated proteins in the HUVECs from the co-culture were gradually increased with increasing oxLDL exposure time, whereas the levels of polyubiquitinated proteins in THP-1 cells were reduced (Figure 4A). Under impaired autophagy conditions induced by oxLDL exposure, the proteasomal pathway, instead of the lysosomal pathway, played an alternative, or supportive, role in clearing cargo. Changes in E3 ligases were assessed at the mRNA level using qPCR. The expression of Cul4, a member of the CRL E3 ligase family, was increased at 24 h and decreased at 48 h after oxLDL exposure in the HUVECs (Figure 4B). In addition, mRNA levels were reduced in p62-silenced HUVECs. Another E3 ligase, NEDD4, is a component of the HECT E3 ligase and its mRNA levels did not change with oxLDL treatment or p62 knockdown (Figure 4C). To further elucidate the role of autophagy and the proteasome-ubiquitin system in clearing atherogenic cargo, p62-siRNA, MG132 (proteasome inhibitor), heclin (HECT E3 ligase inhibitor), and MLN4924 (CRL E3 ligase inhibitor) were administered to cells exposed to oxLDL (Figure 4D). By silencing p62, polyubiquitinated proteins detected by FK2 were abundant, compared to si-control-treated HUVECs. Conversely, the polyubiquitinated proteins were reduced by treatment with MG132 and MLN4924, but were not changed by treatment with Heclin, a HECT E3 ligase inhibitor (Figure 4D).

## 3. Discussion

Atherosclerosis is a chronic inflammatory disease that leads to the development of arterial thrombus. C1q, known to activate the complement cascade, can bind to oxLDL. C1q regulates chemokine production from macrophages during atherosclerosis, so we primarily evaluated its levels in our cell culture model. By accumulating lipoproteins upon oxLDL (50 µg/mL) exposure, secreted C1q levels in the co-culture media were increased 24 h after oxLDL exposure but were not maintained by 48 h (Figure 1). Correspondingly, the protein levels of C1q were increased in co-cultured HUVECs, which triggered inflammation in the THP-1 cells, as determined by detectable cleaved IL-1β protein levels (Figure 1). Primarily, the inflammatory condition was induced by oxLDL exposure in co-cultured HUVECs and THP-1 cells. Additionally, the protein expression levels of C1q and IL-1b in each single-cultured HUVECs and THP-1 cells were a similar pattern to the results of co-culture (Supplementary Figure S1). Under the condition of co-cultured HUVECs and THP-1 cells, the level of C1q and IL-1b was tended to enhance slightly. We investigated the lipid deposition induced by oxLDL via BODIPY staining, as shown in Figure 1. In both the HUVECs and THP-1 cells, oxLDL increased lipid accumulation in a time-dependent manner. Lipid accumulation was detected in ECs by prolonged exposure to oxLDL, as lipids were observed at least 24 h later. Selective receptors of the autophagy pathway participate in innate inflammation; Tax1BP1 and p62 have been reported to mediate the delivery of TRIF and downstream signaling in macrophages [26].

Autophagy is generally recognized as a survival mechanism under starvation and not as a death pathway [27]. In atherosclerotic plaques, autophagy most likely plays a protective role for plaque cells against oxidative stress, a hallmark of advanced atherosclerotic lesions. The results of our autophagy assays showed that autophagy was initiated by oxLDL and was induced earlier in the THP-1 cells than in the HUVECs (Figure 2). In macrophages exposed to oxLDL, autophagy initiation was maintained, indicated by an increased level of beclin-1, and the LC3-II levels were augmented, suggesting that autophagosomes were formed (Figure 2). However, the protein levels of p62 were increased, suggesting autophagy malfunction. The pattern in the THP-1 cells was similar to that in the HUVECs exposed to oxLDL, but the levels of p62 and LC3-II tended to diminish over time (Figure 2). Successful autophagy in atherosclerotic plaques is anti-apoptotic and eventually contributes to cellular recovery. However, under acute or persistent oxidative stress, it can be detrimental. In such a case, the overproduction of intracellular ROS can be harmful to the lysosomal membrane. When autophagy malfunctions during the oxidative stress response in atherosclerotic plaques, or when oxidative injury overwhelms cellular defenses, cells may undergo apoptosis. These results were shown in our previous report in aged human aortic endothelial cells [28]. In the present study, a lysosomal-associated membrane protein, LAMP2, was inversely correlated with p62 and beclin-1 protein expression (Figure 2). This suggests that autophagy was triggered by oxLDL but may be hampered in cells with prolonged exposure to oxLDL. Reducing the LAMP2 expression levels may disrupt the lysosomal degradation in cells under atherosclerotic conditions. In atherosclerosis-disturbed autophagy, the clearing of damaged lysosomes may be impaired, which aggravates atherosclerosis progression [5]. The precise mechanisms by which oxLDL inhibits autophagy in the ECs are unclear. In this study, we evaluated the regulation of autophagy in ECs exposed to oxLDL.

Along with the protein expression levels of p62 and LAMP2, as shown in Figure 2, the mRNA expression levels showed a similar pattern (Figure 3). LAMP2 is crucial for lysosomal fusion with autophagosomes to degrade cellular cargo [29]. Deficiencies in LAMP2 levels, and, thus, the autophagy-lysosomal system, lead to autophagy and vascular homeostasis impairments, resulting in disease progression, including atherosclerosis and cardiac defects [29]. By treating the ECs with siRNA upon oxLDL exposure, we confirmed the downregulation of p62 at the mRNA level; however, LAMP2 levels were not affected (Figure 2). When p62 was downregulated by siRNA, the LC3-II levels were reduced, compared to the si-control-treated ECs upon oxLDL exposure. The downregulation of p62 enhanced the protein levels of LAMP2, which might help alleviate the hampered autophagy and hinder lipid accumulation in the ECs. Lipid accumulation in the cells accelerates the development of atherosclerosis [30], and further studies investigating this process are needed. This study explored the regulation of accumulated lipids by analyzing autophagy in ECs co-cultured with THP-1 cells during exposure to oxLDL. BODIPY staining and immunocytochemistry showed that the lipid accumulation in the HUVECs (Figure 3) and THP-1 cells (Supplementary Figure S2) was reduced, compared to that in the si-control treated cells. Furthermore, the increased p62 did not co-localize with BODIPY, implying that autophagy was not effective. We also examined EC autophagy by assessing LC3 puncta and LAMP2 signals in p62-downregulated ECs. The LAMP2 signals were significantly enhanced, but LC3-positive signals were diminished in si-p62 treated ECs, compared to si-control-treated ECs. It is possible that the downregulation of p62 led to an increase in the LAMP2 levels, but the increased levels of LAMP2 were not involved in autophagy-lysosomal degradation of the lipids.

Under oxLDL exposure, p62 binds polyubiquitinated cargo. Levels of polyubiquitinated proteins and not free ubiquitin (western blotting with FK2 antibody) were enhanced in HUVECs in a time-dependent manner after oxLDL exposure; however, the levels of polyubiquitinated proteins were time-dependently diminished in THP-1 cells (Figure 4). To further evaluate the proteasomal function after prolonged exposure to oxLDL, when autophagy was disrupted, the levels of ubiquitin ligases were measured. The ubiquitin ligase TRAF6 mediates the ubiquitination of ULK1 at K63 and regulates ULK1 stability and activity [31]. Our study assessed Cul4 (a member of the CRL E3 ligase family) and NEDD4 (a member of the HECT E3 ligase family) levels. The Cul4 E3 ligase mediates beclin-1 ubiquitination at K63, and AMBRA1 participates in this process as a substrate adaptor [32]. NEDD4, in contrast to AMBRA1, ubiquitinates beclin-1 at K11 and inhibits autophagy via the proteasomal degradation of beclin-1 [33]. According to our results, Cul4, but not NEDD4, was modulated by the exposure to oxLDL in HUVECs. Additionally, Cul4 expression in HUVECs was regulated by the downregulation of p62 (Figure 4). Since autophagy and the ubiquitin-proteasome system (UPS) interact during protein quality control, or the degradation of intracellular materials, they maintain reciprocal proteostasis [34]. Inhibiting UPS enhances the mRNA levels of p62 and increases the accumulation of ubiquitinated cellular cargo [21,35]. However, the overexpression of p62 does not affect proteasome catalytic activity but leads to ubiquitinated cargo accumulation, which suggests that p62 is not necessary for all types of ubiquitinated aggregates [36]. Thus, p62 has been implicated in many diseases, including cancer [37] and familial amyotrophic lateral sclerosis [19,38]. To inhibit the proteasome and E3 ligases, Cul4 inhibition by MLN4924 treatment increased the LAMP2 protein levels and augmented LC3-II. Each E3 ligase inhibitor did not decrease the levels of polyubiquitinated proteins, whereas proteasome inhibition by MG132 significantly reduced the levels of polyubiquitinated proteins.

Collectively, this study explored p62-mediated autophagy in human ECs under co-culture conditions with macrophages. Upon exposure to oxLDL, lipids accumulated in the ECs and induced autophagy but failed to be degraded after prolonged exposure to oxLDL. Overloaded intracellular lipids cause lysosomal dysfunction, and the proteasomal system can help to alleviate this. This study demonstrates an interaction between lysosomal and proteasomal degradation under atherogenic conditions, and further studies are needed to evaluate the targets of E3 ligase inhibitors, the role of each E3 ligase, and the inter-regulating mechanism between the particular E3 ligases and autophagy-related molecules.

## 4. Materials and Methods

### 4.1. Cell Culture and Drugs

Two cell lines were used in this study. Human umbilical vein endothelial cells (HUVEC) were purchased from Lonza (Basel, Switzerland) and cultured in endothelial growth media EGM-2, as recommended by the manufacturer (Lonza, Switzerland). The human monocytic THP-1 cell line was purchased from American Type Culture Collection (ATCC, Manassas, VA, USA) and cultured in RPMI1640 medium containing 10% FBS and penicillin-streptomycin (100 units/mL and 100 μg/mL, resp.) at 37 °C in a humidified 5% CO_2_ incubator. To mimic the atherosclerotic cellular condition, two cells were co-cultured using an insert (SPL Life Science, Pocheon, Kyonggi-do, Korea) multi-well plate under the exposure of 50 µg/mL oxLDL (Athens Research & Technology, Athens, GA, USA). Three kinds of inhibitors were used for this study: MG132 (Sigma Aldrich, St. Louis, MJ, USA) for proteasome inhibition, Heclin (Tocris Bioscience, Bristol, UK) for HECT E3 ligase inhibition, and MLN4924 (Tocris, UK) for CRL E3 ligase inhibition.

### 4.2. RNA Silencing

A siRNA against p62 (three kinds (20 µM each), OriGene, Rockville, MD, USA) and control-siRNA were independently mixed with siTran 2.0 (OriGene), following the manufacturer’s protocol. The co-culturing THP-1 and HUVECs were then treated with individual siRNAs for 48 h.

### 4.3. Quantitative Real-Time PCR (qPCR) Analysis

Total RNA of the cells (THP-1 and HUVEC) was extracted using a HiGene Total RNA prep kit (BIOFACT Co. Ltd., Daejeon, Korea). Using a One-Step SYBR PrimeScript RT-PCR kit (TaKaRa Bio Inc., Shiga, Japan), RT-qPCR was performed on a QS3 real-time PCR system (Applied Biosystems, Waltham, MA, USA). The PCR amplification was performed for 40 cycles, with denaturation at 95 °C for 5 sec, and annealing and extension at 60 °C for 60 sec. Each sample was performed in triplicates, including no template control, and the results were subsequently analyzed. The differential expression levels were analyzed using the 2–∆∆Ct method and expressed as relative quantity (RQ). The primer sequences for each gene were as follows: human p62 forward, 5′-TGTGTAGCGTCTGCGAGGGAAA-3′; reverse, 5′-AGTGTCCGTGTTTCACCTTCCG-3′; human LAMP-2 forward, 5′-GGCAATGATACTTGTCTGCTGGC-3′; reverse, 5′-GTAGAGCAGTGTGAGAACGGCA-3′; human NEDD4 forward, 5′-CAGAAGAGGCAGCTTACAAGCC-3′; reverse, 5′-CTTCCCAACCTGGTGGTAATCC-3′; human CUL4 forward, 5′-GAATGAGCGGTTCGTCAACCTG-3′; reverse, 5′-CTGTGGCTTCTTTGTTGCCTGC-3′; human β-actin forward 5′-CACCATTGGCAATGAGCGGTTC-3′; and reverse 5′-AGGTCTTTGCGGATGTCCACGT-3′.

### 4.4. Enzyme-Linked Immunosorbent Assay (ELISA)

The secreted level of human C1q in the co-cultured cell culture supernatants was evaluated using a Complement C1q human ELISA kit (Abcam, Cambridge, MA, USA), according to the manufacturer’s instructions. Each culture supernatant loaded plate was incubated for 2 h. After which, the plates were washed and added biotinylated C1q for 1 h. The plate was washed, and streptavidin-HRP was added. Finally, substrate solution was added, and the absorbance was read at 450 nm.

### 4.5. Autophagy Assay

To quantify the autophagy of the cells, the CYTO-ID Autophagy Detection Kit 2.0 (Enzo, Farmingdale, NY, USA) was used, and the experiment was performed via the manufacturer’s protocol. The kit detected the 488 nm-excitable green fluorescence, by reagent supplied in the kit, which is the marker for autolysosomes and earlier autophagic compartments. After treatment under oxLDL, the prepared cells were stained with the Green Detection Reagent 2 in the kit. As a positive control, Rapamycin (0.5 μM) and Chloroquine (10 μM) were treated on the cells, and the cells were additionally cultured for 24 h to induce autophagy. Autophagic cells were analyzed using flow cytometry, and each autophagic cell was shown in percentage.

### 4.6. BODIPY Staining

Each treated cell was incubated on the collagen-coated coverslip for 18 h prior to staining. The cells were washed with PBS, and BODIPY (8 uM) was added to the cells for 30 min at 37°C. After the cells were washed, they were fixed with 2% PFA for 20 min at room temperature. Each cell was washed with PBS three times and then mounted with Vectashield containing 4′, 6-diamidino-2-phenylindole (DAPI) (Vector Laboratory, Carlsbad, CA, USA). The images were obtained using a Zeiss LSM 710 confocal microscope (Carl Zeiss, Thornwood, NY, USA).

### 4.7. Immunocytochemistry

The cells used in this study were plated on the multi-well plate containing coverslip 24 h prior to each treatment and oxLDL exposure (50 µg/mL). After the appropriate treatment and oxLDL exposure, the cells were washed with ice-cold PBS. Cells were fixed in 4% PFA for 20 min and permeabilized with 1% TritonX-100 in PBS. The prepared cells were blocked in 3% BSA in PBS for 1 h and then incubated with each primary antibody overnight at 4 °C as follows: anti-rabbit p62 (1:100, Abcam), anti-mouse LAMP2 (1:100, SCBT), and anti-rabbit LC3II (1:100, Cell signaling biotechnology). Then, each sample was incubated with fluorescence-conjugated secondary antibodies for 1 h at room temperature. We used fluorescein isothiocyanate (FITC)-conjugated donkey anti-rabbit (1:200, Jackson Immunoresearch, West Grove, PA, USA) or rhodamine-conjugated goat anti-mouse (1:200, Jackson Immunoresearch). Each sample was washed with PBS three times for 3 min each and then mounted with Vectashield containing DAPI (Vector Laboratory, Carlsbad, CA, USA) and collected images using a Zeiss LSM 710 confocal microscope (Carl Zeiss, Thornwood, NY, USA).

### 4.8. Western Blot Analysis

Cells were washed in ice-cold PBS and harvested. The collected cells were lysed in a M-er mammalian protein extraction reagent (Thermofisher, Waltham, MA, USA), supplemented with protease and phosphatase inhibitors (Protein Halts, TheroFisher, USA) for 30 min on ice. The equal amounts of protein were loaded and separated on a 4–12% SDS-polyacrylamide gel and transferred onto a polyvinylidene difluoride membrane (PVDF, Millipore, Darmstadt, Germany). After the blocking step, each blot was probed overnight at 4 °C with specific primary antibodies as follows: p62/SQSTM1 (1:1000, Cell Signaling Biotechnology, Danvers, MA, USA), Beclin-1 (1:1000, Cell Signaling Biotechnology), LC3-I/II (1:1000, Cell Signaling Biotechnology), LAMP2A (1:500, Santa Cruz Biotechnology, Dallas, TX, USA), and FK2 (1:1000, Millipore, Darmstadt, Germany). Each blot was then incubated with a HRP conjugated anti-mouse or -rabbit IgG antibodies (Jackson ImmunoResearch, West Grove, PA, USA) for 1 h at room temperature. For internal loading control, blots were probed with HRP-conjugated β-actin (1:2000, Santa Cruz Biotechnology, Dallas, TX, USA). Bands were detected by chemiluminescence (ECL, Pierce, Dallas, TX, USA) and visualized on LAS 4000 (Fujifilm, Tokyo, Japan).

### 4.9. Statistical Analysis

Data are presented as mean ± standard deviation (SD) and analyzed using GraphPad Prism software (version 7.01). Statistical analyses were performed using unpaired t-test and one-way ANOVA. Differences were considered to be statistically significant at * *p* < 0.05, ** *p* < 0.001, and *** *p* < 0.0001.

## Data Availability

Not applicable.

## Supplementary Materials

Supplementary materials can be found at https://www.mdpi.com/article/10.3390/ijms22157791/s1.
