# Supplementary material for: P62 Links the Autophagy Pathway and the Ubiquitin–Proteasome System in Endothelial Cells during Atherosclerosis
Source: Int J Mol Sci. 2021 Jul 21;22(15):7791. doi: 10.3390/ijms22157791 (PMC8346161; doi:10.3390/ijms22157791)
Supplement: Supplementary file 1 [file ijms-22-07791-s001.zip › ijms-1298919-supplementary.pdf]

Supplementary Materials

P62 Links the Autophagy Pathway and the Ubiquitin–Proteasome System in Endothelial Cells during Atherosclerosis

Sejeong Kim [1,2], Woong-Jin Lee [2,3] and KyoungJoo Cho [4,*]

[1] College of Korean Medicine, Sangji University, Wonju 26339, Korea; nehemier@nate.com
[2] Department of Cognitive Science, Yonsei University, Seoul 03722, Korea; osang0616@yuhs.ac
[3] Department of Neurology, College of Medicine, Yonsei University, Seoul 03722, Korea
[4] Department of Life Science, Kyonggi University, Suwon 16227, Korea
* Correspondence: kcho0611@kgu.ac.kr; Tel: +82-31-429-1365

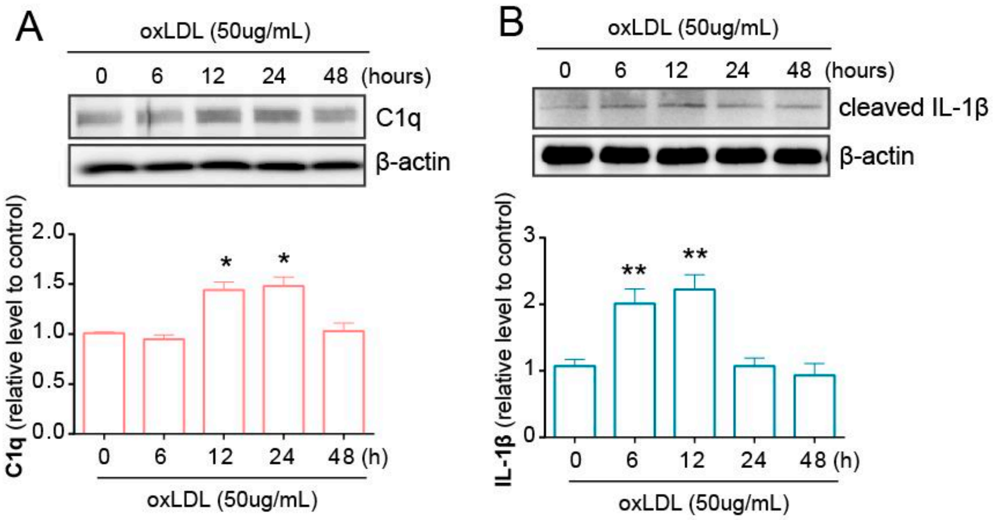

Figure S1. Exposure of oxLDL on each single-cultured macrophages and endothelial cells at several time points after oxLDL exposure. (**A**) HUVECs and (**B**) THP-1.

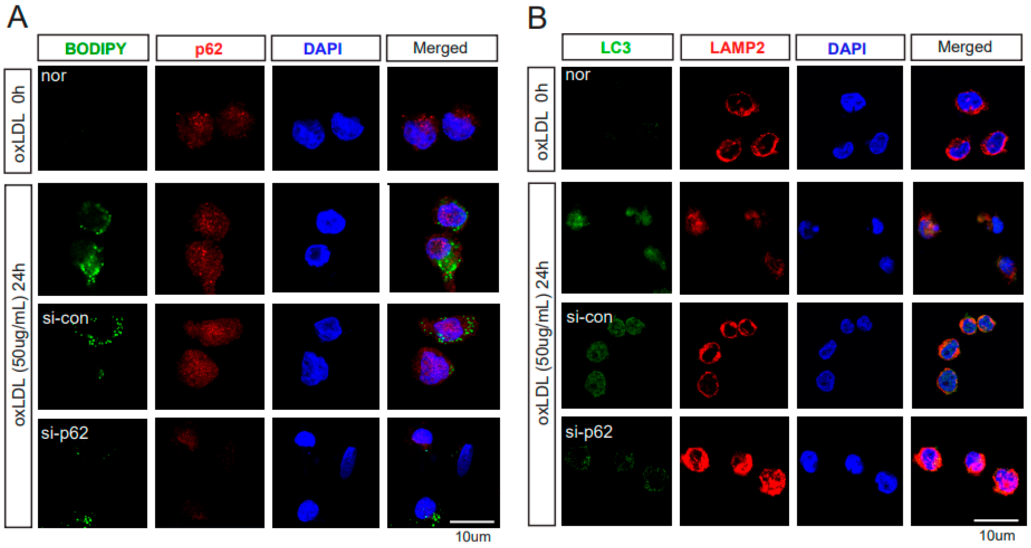

Figure S2. Immunocytochemistry in THP-1 cells. **(A)** After oxLDL exposure, accumulated lipids and p62 were detected in THP-1 cells after treatment with si-p62 for 24 h and **(B)** LC3 and LAMP2 were detected in si-p62 treated THP-1 cells upon oxLDL exposure.
